# Epithelioid Hemangioendothelioma of Nasal Cavity

**DOI:** 10.4103/0974-2727.72214

**Published:** 2010

**Authors:** Rashmi Patnayak, Amitabh Jena, M Kumaraswamy Reddy, Amit kumar Chowhan, LM Chandrasekhar Rao, N Rukhamangadha

**Affiliations:** Department of Pathology, Sri Venketeswar Institute of Medical Sciences, Tirupati, Andhra Pradesh, India; 1 Department of Surgical Oncology, Sri Venketeswar Institute of Medical Sciences, Tirupati, Andhra Pradesh, India

**Keywords:** Epithelioid hemangioendothelioma, nasal cavity, immunohistochemistry

## Abstract

We present a rare case of epithelioid hemangioendothelioma (EHE) in the nasal cavity of a 40-year-old Indian male who presented with history of intermittent epistaxis. The lesion was tested for a panel of immunohistochemical markers like vimentin, CD31, CD34, Factor VIII, vascular endothelial growth factor (VEGF) and Ki67. Immunohistochemically, the neoplasm showed striking positivity for vimentin, CD31, CD34 and weak positivity for VEGF; positivity was also noted for Factor VIII especially in the miniature intracytoplasmic vascular lumina.

## INTRODUCTION

Epithelioid hemangioendothelioma (EHE) is a rare vascular tumor, which was described first in 1982 by Weiss and Enzinger.[1] This unusual vascular neoplasm is characterized by proliferation of a distinct type of endothelial cells which exhibit epithelioid morphology. This is an indolent tumor with intermediate or borderline malignant potential as the tumor is potentially recurrent, but rarely metastasizes.[2] There are very few cases of EHE of nasal cavity described in the English literature.[3] We hereby describe an EHE of nasal cavity along with its immunohistochemical findings.

## CASE REPORT

**Figure 1:**
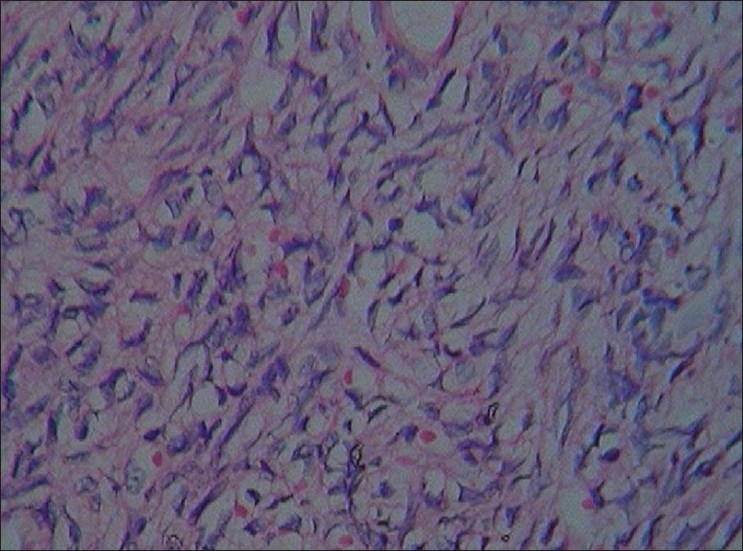
Lesion composed of plump cells with large nuclei showing intracytoplasmic lumen containing red blood cells (hematoxylin and eosin staining, ×400)

A 40-year-old Indian male presented with 2 months history of a slowly growing firm swelling in the nasal septum associated with intermittent epistaxis. The patient’s medical history was not significant. The routine general examination was also within normal limits. Grossly, the excised lesional mass was globular with cut section showing capsulated grayish white solid mass of 1 × 1 cm size. Histologically, the hematoxylin–eosin (H–E) stained section showed surface pseudostratified ciliated columnar epithelium, thin fibrous encapsulation and presence of seromucinous glands both within and outside the lesional component. The lesion proper comprised epithelioid plump to spindle cells, with large vesicular nuclei and distinct cell border, mild acidophilic to clear cytoplasm, mild to moderate nuclear pleomorphism and scattered mitotic figures. The neoplastic cells were forming miniature capillary buds or vascular channels with little cytoplasmic lumen and many of them were containing red blood cells [Figure 1]. Areas of hyalinization and edema were also seen in the stroma. There was no tumor necrosis. Based on these findings, the histopathological diagnosis was made as EHE.

**Figure 2:**
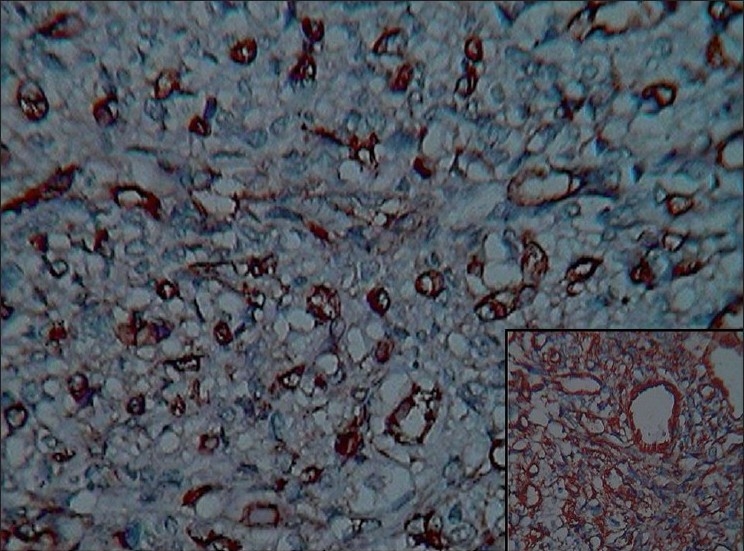
Positivity for CD34 and CD31 (inset) (immunohistochemical staining, ×400)

Reticulin staining as well as a panel of immunohistochemical markers were performed to confirm the diagnosis. The reticulin staining highlighted a dense reticulin network surrounding individual tumor cells. Immunohistochemically, the tumor was positive for vimentin, CD31, CD 34 [Figure 2] and also for anti-factor VIII. This vascular marker was more accentuated at the site of miniature intracytoplasmic vascular lumina. Immunostain for Ki67, a proliferation marker, showed only 10% nuclear positivity and, in addition, weak staining of vascular endothelial growth factor (VEGF) was noted.

## DISCUSSION

EHE in the head and neck region is extremely rare. The common locations of EHE include soft tissue of extremities, skin, liver, lung and bone. Very few cases of EHE are described in nasal cavity in the current literature.

Among the vascular tumors of nasal cavity, lobular capillary hemangioma is quite common. This circumscribed lesion is composed of lobules of capillaries lined by plump endothelial cells. The stroma in between the lobules is fibromyxoid. Though high mitoses may be observed in the cellular areas, atypical ones are not seen yet.

Glomangiopericytoma (Sinonasal hemangiopericytoma) has predilection for the nasal cavity and paranasal sinuses. This unencapsulated, cellular tumor is usually well-delineated. It comprises elongated to oval neoplastic cells forming short fascicles and occasional ones even exhibiting storiform pattern. Many interspersed vascular channels with “staghorn” or “antler- like” configuration are seen. Characteristically, they exhibit peritheliomatous hyalinization. Though mild nuclear pleomorphism and occasional mitotic figures may be seen, necrosis is distinctly uncommon.[4]

In contrast to the above vascular tumors, EHE is composed of a distinctive type of endothelial cells with abundant eosinophilic cytoplasm and many intracellular vascular lumina producing cytoplasmic vacuolations. There are usually minimal mitoses and pleomorphism.[5] Histologically, the lobular pattern of lobular capillary hemangioma and the staghorn blood vessels, peritheliomatous hyalinization of glomangiopericytoma are not appreciated in EHE.

EHE is a low-grade malignancy. The poor expression of proliferation marker Ki67 and weak positivity for VEGF, a dimeric polypeptide growth factor known for its specificity for vascular endothelial cells, are indication of the less aggressive nature of the lesion.[5,6] On occasions, EHE can be misdiagnosed as carcinoma on account of its remarkable cellularity and the mitotic activity.[1] In those cases, the positive staining for factor VIII is helpful in differentiating EHE from carcinoma.[5] EHE has a tendency for local recurrence and regional lymphnode metastasis. Treatment of choice for localized disease is complete excision with negative margins. Since some of the incompletely or inadequately excised tumors, especially tumors located in the head and neck region, with undefined boundaries, exhibit multiple recurrences, radiation therapy can be considered in those patients. Anti-angiogenic agents also may play a role in the treatment of these vascular tumors in future. This tumor needs long-term follow-up because of its propensity for recurrence. In addition, clinically lymph nodes should be evaluated to exclude rare instance of lymphnodal metastases. The histopathological findings in correlation with immunohistochemistry help in the definitive diagnosis of this tumor.

Our patient is symptom free, 9 months after resection of the tumor. This is an additional case of EHE highlighting the immunohistochemical features and to corroborate the borderline malignant nature of the lesion.
